# Liquid biopsy can cure early colorectal cancer recurrence – Case Report

**DOI:** 10.3389/fonc.2023.1141833

**Published:** 2023-05-03

**Authors:** Alexander Baraniskin, Hideo A. Baba, Dirk Theegarten, Thomas Mika, Roland Schroers, Susanne Klein-Scory

**Affiliations:** ^1^ Department of Hematology and Oncology, Evangelisches Krankenhaus Hamm, Hamm, Germany; ^2^ Department of Medicine, Hematology and Oncology, University Hospital Knappschaftskrankenhaus Bochum GmbH, Ruhr University of Bochum, Bochum, Germany; ^3^ Institute of Pathology, University Hospital Essen, University Duisburg-Essen, Essen, Germany

**Keywords:** liquid biopsy, ddPCR, colorectal cancer, follow-up, Ras, tumor promotor P53 TP53, mutation detection, PET-CT

## Abstract

In the context of colorectal cancer (CRC), circulating tumor DNA (ctDNA) is frequently used to monitor the minimal residual disease (MRD). ctDNA has become an excellent biomarker to predict which patients with CRC are likely to relapse due to the persistence of micrometastases. MRD diagnosis *via* analysis of ctDNA may allow much earlier detection of relapse compared with conventional diagnosis during follow-up. It should lead to an increased rate of curative-intended complete resection of an asymptomatic relapse. Besides, ctDNA can provide crucial information on whether and how intensively adjuvant or additive therapy should be administered. In the present case, analysis of ctDNA gave us a crucial hint to the use of more intensive diagnostics (MRI and Positron emission tomography–computed tomography PET-CT) which led to earlier detection of CRC relapse. Metastasis detected early are more likely to be completely resectable with curative intent.

## Introduction

Despite advances in diagnostic imaging, surgery, and chemotherapy, the 5-year mortality rate of colorectal cancer (CRC) remains high with nearly 40% (1). The key for successful CRC treatment is early detection, as the 5-year survival rate at stages I and II is above 80%, but after the development of distant metastases, it decreases to approximately 10% (2). The primary goal of curative CRC therapy is complete resection of the tumor tissues combined with adjuvant chemotherapy in advanced situations. Identifying patients with minimal residual disease (MRD), i.e. clinically hidden micrometastases remaining after initial therapy, and treating with additional or intensified therapy could potentially increase the rate of cured patients.

One limiting factor in MRD diagnostics of CRC is the low sensitivity of standard diagnostical tools including imaging (e.g., Magnetic Resonance Imaging (MRI), Computed Tomography (CT), or Positron emission tomography–computed tomography (PET/CT) and serum tumor markers, e.g. carcinoembryonic antigen (CEA) or cancer antigen 19-9 (CA 19-9) (3). Serial CEA analyses may recognize relapse with a sensitivity of 69% and specificity of 64% (4). Furthermore, detection of ctDNA after precise and complete surgical removal of the tumor can be used to manage the chemotherapy options for patients with CRC stage II and III (5, 6). Only patients with MRD should receive chemotherapy, while 13% of patients could be spared the unnecessary chemotherapy (6, 7). The reduction of the number of chemotherapies results in the same 83% rate of cure as the standard treatment management.

Stage IV CRC is associated with higher likelihood of relapse and poorer survival outcomes. Oligometastatic CRC is eligible for surgery with curative intent, but 60%–70% of patients will go on to relapse postresection (8).

Thus, there is an unmet need for the development of better tools to facilitate physician’s decision-making in identifying and stratifying resected patients by risk of relapse with still curative intent. Strikingly, patients with asymptomatic recurrences reveal a more than 5-fold higher 5-year overall survival compared to symptomatic recurrences (9). It is remarkable that nowadays, over 60% are still diagnosed with recurrence secondary to symptoms. Thus, early detection of higher risk of metastasis after tumor resection is crucial for improving clinical outcomes of patients.

In the past few years, blood-based liquid biopsies - especially the analysis of cell-free tumor DNA (ctDNA) - have received widespread attention due to increased sensitivity of modern polymerase chain reaction (PCR)-based technologies. The detection of plasma ctDNA is prognostic in CRC and has the potential to serve as a highly specific and low-invasive test for early prediction of disease recurrence in clinical routine may enable a locoregional approach (5, 6, 10).

In the here reported CRC case, we present the significance of liquid biopsy for MRD diagnostics in advanced CRC.

## Case description

**Figure 1:**
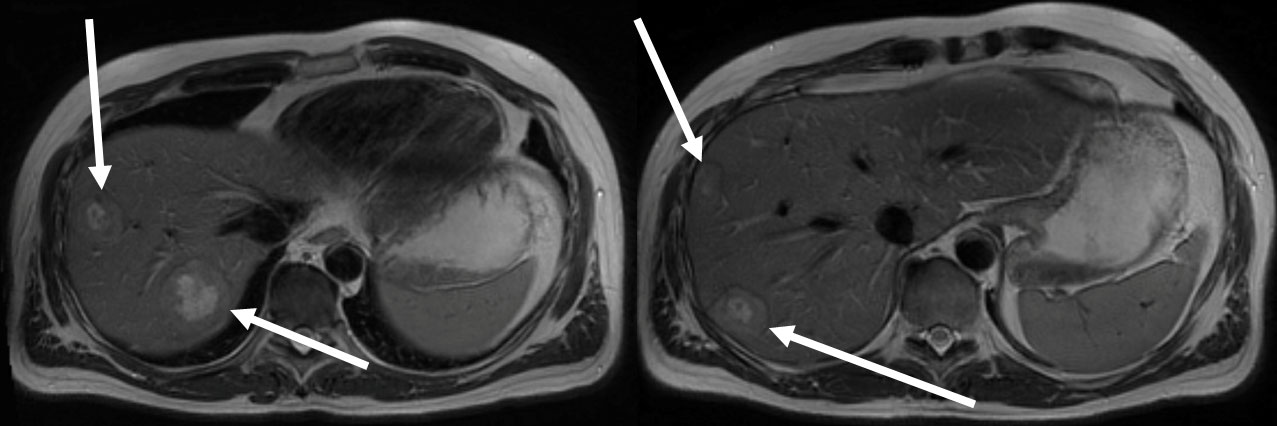
Multiple liver metastases limited to right liver lobe in MRI at diagnosis in September 2019 (white arrows).

A 47-year-old man without any significant pre-existing conditions was diagnosed in September 2019 with a rectum adenocarcinoma (upper rectal) with synchronous liver metastases (limited to right liver lobe) (see the magnet resonance imaging (MRI in **Figure 1**). The molecular diagnostics of tumor tissue revealed mutations in *neuroblastoma RAS viral oncogene homolog* (NRAS2 G12D c.35G>A) and in *tumor protein p53* (TP53 C141Y c.422G>A). A neoadjuvant therapy with four cycles of FOLFOX4 (Folic acid, fluorouracil, and oxiliplatin 24h) and bevacizumab was administered and was well tolerated. In December 2019, the patient underwent exploratory laparoscopy and right hemihepatectomy. Five metastases were removed. By the tumor regression grading (TRG) of 2, the histological response to chemotherapy of hepatic colorectal metastases (HCRM) was grade 4 with 90% of necrosis of surface.

One month later, laparoscopically assisted anterior rectum resection, sigmoid resection and descendorectostomy followed. The TNM stage was ypT2 ypN0(0/17) ypM1 (hep). Thereupon, additive therapy with eight cycles of FOLFOX4 was given and was well tolerated again.

Starting in September 2020, liquid biopsies to detect the known *NRAS* mutation were carried every six months. In July 2021, *NRAS* mutations were not yet detectable.

**Figure 2:**
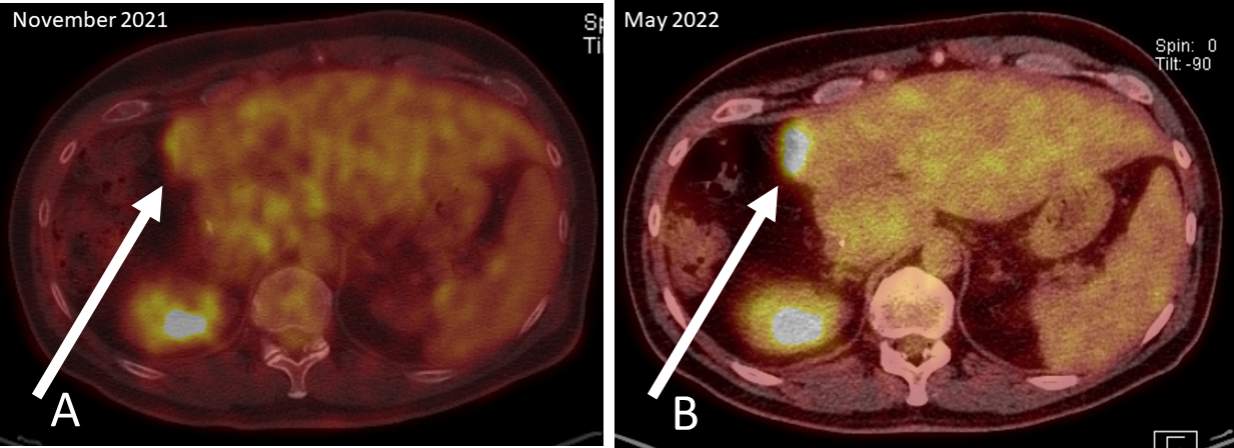
PET-CT scans: **(A)** November 2021; diffusion restriction at the resection margin with albeit minimal FDG activity (white arrow), **(B)** May 2022; clear increase of FDG activity in the resection margin in liver segment IV.

A routine examination for relapse in November 2021 included computed tomography of chest and abdomen, MRI of the liver, sigmoidoscopy and analyses of tumor markers CEA and CA19-9. The MRI showed an annular contrast medium enhancement at the resection margin in liver segment IV. To further classification of the contrast medium enhancement, subsequently conducted 2-Deoxy-2-[18F]fluoro-d-glucose emission tomography (FDG-PET-CT) showed diffusion restriction at the resection margin with albeit minimal FDG activity (white arrow; Standardized uptake value (SUV) max 4.3), which fell within the range of the liver parenchyma (SUV 1.9-4.8) (See PET-CT scan in **Figure 2A**). Overall, the examinations were assessed as not malignancy-suspect.

**Figure 3:**
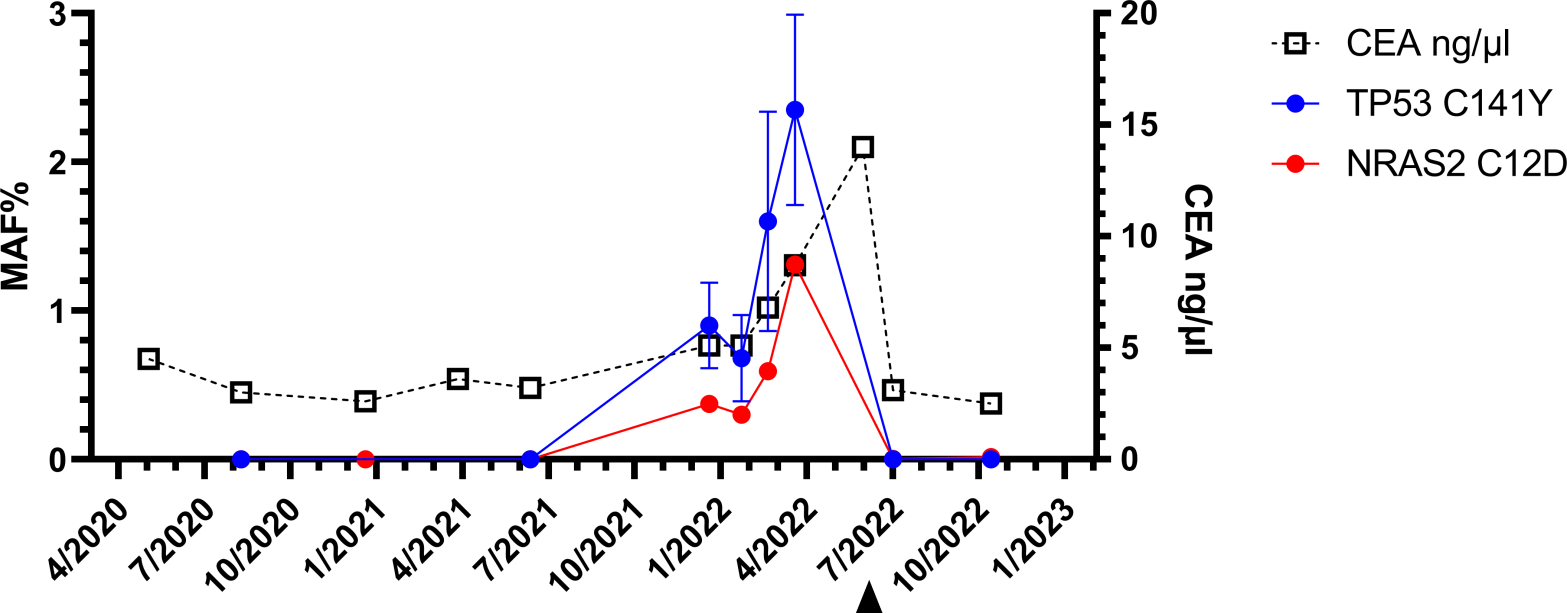
Longitudinal liquid biopsy results: The first three liquid biopsy analyses until the 15th month of the follow up were negative. The fourth sample revealed *NRAS* (red)- and *TP53*-(circles blue) mutations. Only after the resection (arrowhead), the ctDNA was no longer detectable. Three months after the reappearance of the *NRAS2 C12D* mutations, the CEA level also increased substantially.

However, five weeks later (January 2022), both, the original *NRAS* and *TP53* mutations were detectable with mutant allele fractions (MAF) of 0.37% and 0.9% respectively (**Figure 3**).

An intensified search for a relapse was undertaken since January 2022. CT scans, MRI of the liver and the tumor markers showed unchanged findings and an assessment that no metastasis exist. The next liquid biopsy analysis in March 2022 and April 2022 continued to show increasing MAF for both mutations. Based on liquid biopsy result, a premature PET-CT was performed in May 2022. This PET-CT revealed a clear increase of FDG activity in the resection margin in liver segment IV (See PET-CT scan in **Figure 2B**). The curative-intent complete resection of asymptomatic relapse followed. The tissue analysis of resected material confirmed the *NRAS* and *TP53* mutations detected in liquid biopsy and measured the *NRAS2* mutation with an allele frequency of 47.4% and the *TP53* mutation with 68.4%. The metastasis tissue was graded as G2 with 50% necrotic parts. No hint for microsatellite instability was found. The postoperative liquid biopsy samples no longer contained ctDNA with *NRAS* or *TP53* mutations. The tumor marker carcinoembryonic antigene CEA was not substantially elevated until March 2022 and decreased to normal after surgery in July 2022 (**Figure 3**). We have no signs of a recurrence so far (till February 2023). The patient is symptom-free, works full-time and leads a normal family life.

## Discussion

We report the clinical course of a patient in follow-up after resection of rectum carcinoma and hepatic metastases. Longitudinal liquid biopsies revealed MRD with detection of *NRAS* and *TP53* mutations 4 month earlier as detected by PET-CT. Only by intensifying the search for disease recurrence on the basis of ctDNA detection, the location of CRC relapse could be unraveled.

MRD-positivity in liquid biopsy displays without ifs and buts an overwhelming suspicion of a persistent disease. Several studies have demonstrated that patients with detectable ctDNA postsurgery will finally develop a relapse (4, 5, 11). In patients diagnosed with resected stage I-III CRC, MRD-positivity at the end-of-treatment (surgery with/without adjuvant chemotherapy) was accompanied by an over 40 times higher probability of disease recurrence as compared to MRD-negative patients (4, 11). In a study by Tie et al., patients who underwent curative intended resection of colorectal cancer liver metastases were further analyzed. MRD-positivity at the end-of-treatment was associated with a 5-year RFS (recurrence free survival) of only 0% and a 14.9 times higher recurrence probability. In line with our clinical report, longitudinal ctDNA analyses identified disease recurrence up to 16.5 months and in mean 8.7 months earlier than standard radiologic imaging (4).

In our clinical report, the knowledge of the MRD-positivity influenced the intensity and manner of follow up. Based on this finding, the decision to carry out the PET-CT examination was made. This approach may have enabled the curative-intent complete resection of the relapse.

## Methods

Molecular analysis of the tissue samples was routinely performed by next generation sequencing (Institute of Pathology, University Hospital Essen, Germany). Two mutations were found at a 60% cellularity with a proportion of 68.4% MAF of *TP53 C141Y c.422G>A* mutation and 47.4% MAF% of *NRAS2 G12D c.35G>A* mutation.

Blood samples were obtained in cell free DNA collection tubes (Cell-Free DNA BCT; Streck™) and sent to our lab (Ruhr University Bochum, Knappschaftskrankenhaus). Plasma was isolated latest three days after blood collection.

The ctDNA was isolated from 3 ml plasma using the circAMP circulating nucleic acid isolation procedure (Qiagen™, Hilden, Germany) as described earlier (12). NRAS2-12 mutation detections were performed using the IVD certified all RAS mutation kit with ONCOBEAM technology (Sysmex Inostics, Hamburg, Germany).

The assay to detect the mutation *TP53 C141Y c.422G>A p.*C141Y, NM_002524.4 COSM43708 was produced by Bio-RAD (Hercules, California, USA). The context sequence was given in supplemented file (**Supplementary material**). The ddPCR was performed as described in (12).

To validate the ddPCR-based assay to detect the *TP53 C141C* mutation, genomic DNA from the patient´s liver metastasis was used (**Supplementary material**).

The tumor marker carcinoembryonic antigene was measured by routine procedure in clinical labs.

## Data availability statement

The original contributions presented in the study are included in the article/**Supplementary Material**. Further inquiries can be directed to the corresponding author.

## Ethics statement

Ethical review and approval was not required for the study on human participants in accordance with the local legislation and institutional requirements. The patients/participants provided their written informed consent to participate in this study.

## Author contributions

AB and SK-S contributed equally to the writing of the manuscript and designed the figures. AB was involved in the treatment of the patient. AB, DT, HB, RS, SK-S, and TM reviewed and approved the final version of this work. All authors contributed to the article.
