# Supplementary material for: Liquid biopsy can cure early colorectal cancer recurrence – Case Report
Source: Front Oncol. 2023 May 3;13:1141833. doi: 10.3389/fonc.2023.1141833 (PMC10188995; doi:10.3389/fonc.2023.1141833)
Supplement: Supplementary file 1 [file DataSheet_1.pdf]

Supplementary figures

A

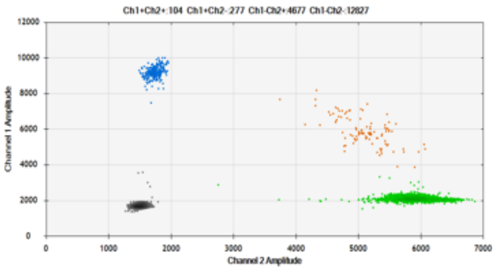

7% MAF positive control

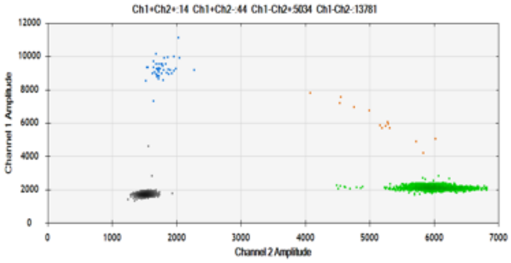

0,7% MAF positive control

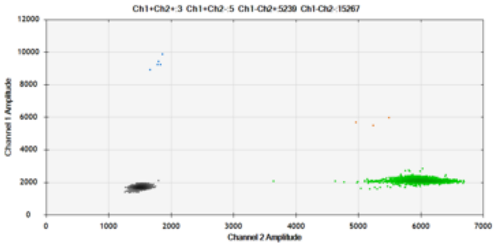

0,07% MAF % positiv contol

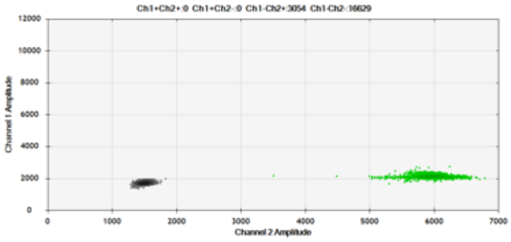

MAF% negative control

B

hg19|chr17:7578447-7578569	Amplicon length
TTCCTCTTCCTACAGTACTCCCCTGCCCTCAACAAGAT<br>GTTTTGCCAACTGGCCAAGACCT[G/A]CCCTGTGCAGCTGTGGG<br>TTGATTCCACACCCCCGCCCGGCACCCGCGTCCGCGCCATGGC<br>C	63<br>nucleotide<br>s

Supplementary figure 1 A Examples of TP53 C141Y ddPCR result: 2D blot of positive control in different SPIKE IN amount in background. B The wild-type and mutant allele variant assays were labelled with HEX and FAM fluorescent dye, respectively. The ddPCR conditions were chosen as described in Klein-Scory et al.2020

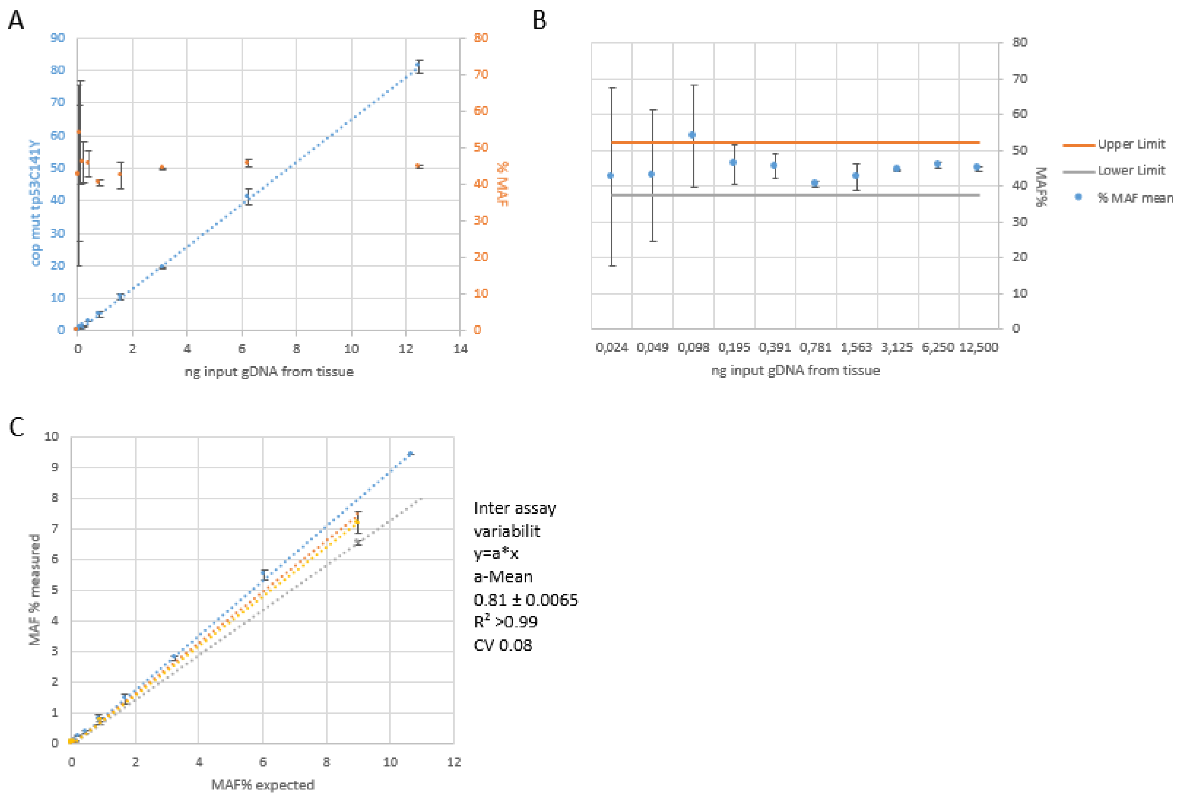

Supplementary figure 2 Validation of the ddPCR TP53 assay . Titration of positive control in TP53 C141Y ddPCR assay without background (A,B) and with background in 4 independent experiments (C). For positive control, the genomic DNA was extracted from 15 sections of formalin-fixed, paraffin embedded tissue from the patient´s liver metastasis. The sections were deparaffinized using the mineral oil method and the gDNA was isolated using Reliaprep kit according to the manufacture´s protocol (Promega Incorp., Walldorf, Germany). This gDNA from the patient´s metastasis was used as reference material to define the precision, accuracy of the ddPCR assay starting from 12.5 ng input gDNA with a dilution series in a ratio of 1:1 till 0.02ng. The MAF% of the gDNA remained constant at 44.9 ± 3.6 (95% confidence interval: 43.4-46.4; CV 0.08) till at least 0.2 ng input DNA (A,B). The linearity of the ddPCR assay was ascertained using the tissue isolated gDNA for titration as a “spike in” in a background of wild-type DNA (C). Limit of detection was estimated with 0.08 MAF%, and a cut off level of 0.2% MAF and more than 2 mutant events.
